# Controlled nanocrystallization of gold nanoclusters within surfactant envelopes: enhancing aggregation-induced emission in solution†

**DOI:** 10.1039/d4sc02834a

**Published:** 2024-06-24

**Authors:** Yuki Saito, Ayano Suda, Maki Sakai, Shogo Nakajima, Yukatsu Shichibu, Hayato Kanai, Yasuhiro Ishida, Katsuaki Konishi

**Affiliations:** a Faculty of Environmental Earth Science, Hokkaido University North 10 West 5 Sapporo 060-0810 Japan konishi@ees.hokudai.ac.jp; b Graduate School of Environmental Science, Hokkaido University North 10 West 5 Sapporo 060-0810 Japan; c RIKEN Center for Emergent Matter Science 2-1 Hirosawa Wako Saitama 351-0198 Japan

## Abstract

The nanocrystallization of functional molecules has been a subject of recent interest in the current development of nanotechnology. Herein, we report the unprecedented synthesis of single nanocrystals of a molecular gold nanocluster in a homogeneous solution by using surfactant-based nano-envelopes. The co-assembling of a Au_8_ nanocluster carrying lipophilic phosphine ligands with sodium dodecyl sulfate (SDS) in an aqueous solution results in the formation of sphere-shaped amorphous nano-aggregates coated with the surfactant. Upon sonication, the spherical amorphous aggregates are smoothly shape-shifted into discrete rhombic nanocrystals, which can be tracked by TEM and solution XRD. The transformation into single nanocrystals occurs exclusively without further growth or agglomeration, implying that the crystal growth is restricted within the surfactant nano-envelopes. The robust but flexible nature of the wrapped surfactant is likely responsible for the controlled crystallization. We also demonstrate that the amorphous-to-nanocrystalline transition in solution remarkably enhances the photoluminescence emission from the nanocluster, providing a clear example of crystallization-induced emission enhancement. Notably, the obtained nanocrystals showed high stability in solution and retained their shape, size, and PL intensity even after several months, owing to the densely packed surfactant shell. The present surfactant-directed nanocrystallization method may be applicable to other molecular species to contribute to the development of nanocluster science as well as the designed synthesis of nanomaterials.

## Abstract 2

Sphere-shaped amorphous aggregates of Au_8_ clusters entrapped within SDS nanoenvelopes are transformed into rhombic single nanocrystals upon simple sonication in solution, where the aggregation-induced emission (AIE) from Au_8_ is notably enhanced.

## Graphical abstract

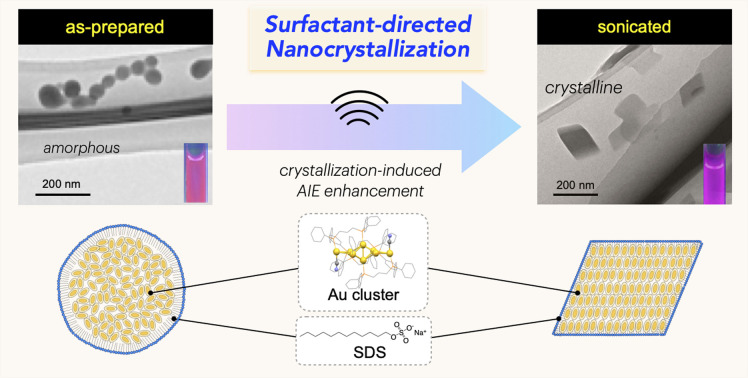

## Introduction

Nanospaces physically segregated from the outer phase have attracted continuing attention because they endow encapsulated substances with unusual reactivities and properties.^1,2^ Self-assembled surfactants such as vesicles and micelles are typical examples providing such isolated inner environments, and it is well known that the accommodation of specific substrates within surfactant envelopes brings about distinctive features that are not achieved in open systems.^3–5^ Self-organization of small molecules within nanospaces is particularly interesting, since the spatial restrictions and characteristic interior surfaces should induce unique arrangements of the entrapped molecules to cause emergent effects.^6–8^

The controlled synthesis of nanocrystals is a continuing topic in current nanotechnology because of their unique properties associated with size effects in the nanoscale regime. To maintain the crystal size at the nm level by preventing uncontrolled growth and/or agglomeration is a central issue, for which the utility of surfactants is expected. The syntheses of inorganic nanocrystals of colloidal metal nanoparticles and metal oxides, whose frameworks are constructed by strong covalent-type bonds, have been actively studied and already reviewed.^9–11^ On one hand, molecular nanocrystals held by weak non-covalent forces have been less studied,^6–8,12–17^ and the establishment of general synthetic protocols is desired. In this paper, we report an example of the nanocrystallization of luminophore molecules by showing clear morphological alteration of surfactant-coated amorphous molecular aggregates into discrete single nanocrystals. The amorphous-to-crystalline transition that occurs in a dispersed homogeneous solution system allows direct comparison of the photoluminescence efficiency in terms of the packing feature.

The luminescent module we used here is a ligand-protected Au_8_ nanocluster with structural precision. Atomically precise coordinated metal nanoclusters have recently emerged as a novel family of functional molecular blocks because of their unique optical/electronic properties and reactivities.^18–21^ Although a number of crystals and crystal structures have been identified for such molecular nanocluster compounds, there have been few reports of nano- or submicron-crystals.^22^ We show that co-assembly of a cationic Au_8_ cluster with sodium dodecyl sulfate (SDS), a representative anionic surfactant, under aqueous conditions leads to the formation of a solution of sphere-shaped amorphous cluster nano-aggregates coated with the surfactant. Upon sonication in solution, the as-synthesized nano-composite spheres are smoothly converted into angularly shaped single nanocrystals, showing an example of “*in situ*” crystallization within the surfactant nano-envelopes. From the results of the elemental mapping analyses and the effects of the initial concentration of the surfactant, we discuss the mechanism of the nanocrystallization accompanying explicit shape shifting and shed light on the robust but flexible nature of the surfactant monolayer wrapping the cluster aggregates. We also highlight that the increase in crystallinity in a homogeneous solution leads to the enhancement of the aggregation-induced photoluminescence emission (AIE) from the gold nanocluster, providing a clear example of crystallization-induced emission enhancement. The findings shown here present an unprecedented example of the solution synthesis of molecular nanocrystals of gold nanoclusters and reveal the utility of surfactant-based inner nanospaces for the controlled synthesis of nanocrystals with unique crystal structures and properties.

## Results and discussion

### SDS-coated amorphous nanoaggregates of gold nanoclusters

**Fig. 1:**
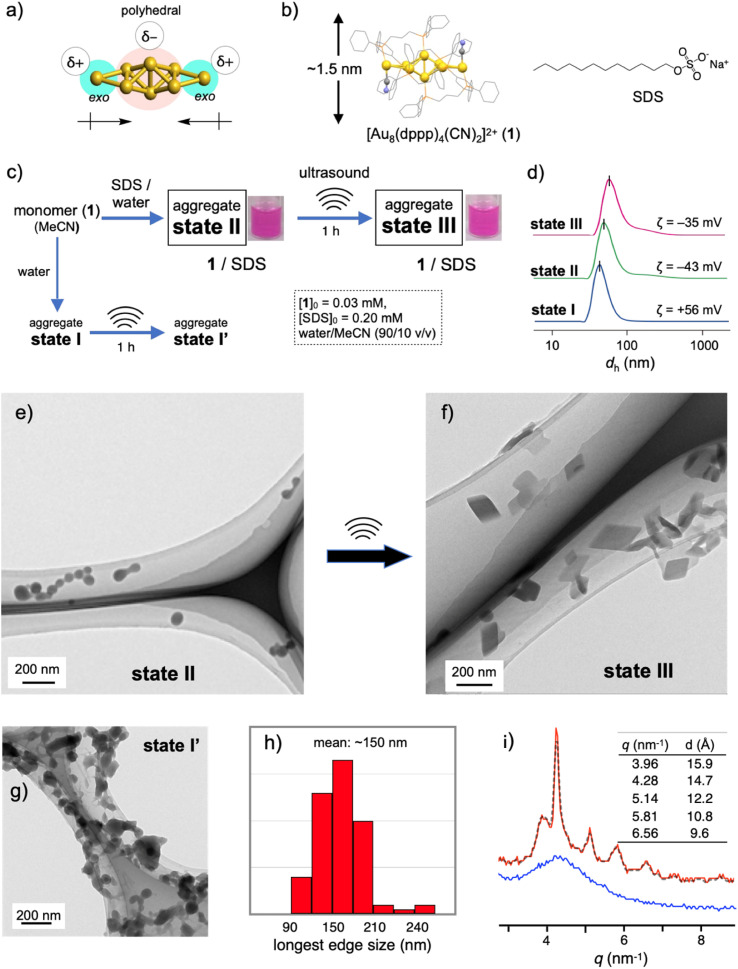
(a) Schematic illustration showing the polar nature of the [core + *exo*]-type Au_8_ cluster. (b) Chemical structures of 1 and SDS. (c) Evolution to several aggregation states of 1. (d) Number-based dynamic light scattering (DLS) profiles of the assembly samples of states I–III. (e–g) TEM images of the assembly samples of states II, III and I′. (h) Distribution of the longest edge lengths of the rhombic objects found in the TEM images of state III. (i) XRD profiles (SPring-8 BL05XU beam line) of solution samples of states II and III in MeCN/water (10/90 v/v).

Recent rapid advances in the field of atomically precise metal clusters have led to the use of various types of gold clusters. Among them, we take notice of gold nanoclusters whose gold frameworks consist of a polyhedral core and additional gold atoms attached outside (Fig. 1a).^23–30^ Previous studies on the aggregation behaviors and optical responses in solution have revealed that such [core + *exo*]-type gold clusters are apt to align spontaneously owing to the inherently polar nature of the gold frameworks.^29,30^ Here we used an octagold cluster [Au_8_(dppp)_4_(CN)_2_]^2+^ (dppp: Ph_2_P(CH_2_)_3_PPh_2_) (1) (Fig. 1b). These clusters (nitrate salt) exist as monomers in anhydrous MeCN but instantly aggregate upon mixing with a large portion of water due to the hydrophobic character of the outer dppp layer. For example, the dynamic light scattering (DLS) profile of the solution of 1 (30 μM) in MeCN/water (10/90 v/v) (state I, Fig. 1c) gave an average hydrodynamic diameter (*d*_h_) of ∼40 nm (Fig. 1d), which is much larger than the single-molecule size (∼1.5 nm, Fig. 1b). Accordingly, the transmission electron microscopy (TEM) image showed large spherical objects (Fig. S2b†), which contrast with those found in the image of the dispersed monomers (a) prepared from the anhydrous MeCN solution. The *ζ*-potential measurement gave a positive value (+56 mV), showing that the nanoobjects are composed of cationic clusters and have a positively charged surface that is spatially segregated from the counter anions. When sodium dodecyl sulfate (SDS) (0.2 mM) was included in this solution (state II, Fig. 1c), the formation of similar spherical objects with a *d*_h_ of ∼50 nm was observed in the DLS and TEM profiles (Fig. 1d and e). However, the *ζ*-potential became negative (−43 mV), implying the formation of giant micelles with a lipophilic cluster aggregate inside and anionic sulfate heads exposed to the outer bulk medium. The diameter of state II was much larger than that expected for conventional single-layer SDS micelles (∼5 nm),^31^ indicating that the formation of giant micelle-like composites is essentially governed by the aggregation of cationic clusters.

### Sonication-induced nanocrystallization inside giant micelles

The 1–SDS nanocomposite in state II, thus obtained, appeared stable, as the above DLS and TEM profiles were retained when the solution was kept at room temperature (∼25 °C) for several days (Fig. S3†). However, when this composite solution (state II) was sonicated for 1 h in a conventional ultrasonic water bath (38 kHz, 100 W; bath temp. 25–35 °C) (state III, Fig. 1c), drastic alterations in the nanoscale morphology were observed in the TEM images. Typically, as shown in Fig. 1f, the spherical objects found before sonication (state II, Fig. 1e) completely disappeared, and instead, rhombic (parallelogram-shaped) angular objects that look like single crystals appeared. Upon shape shifting, the average hydrodynamic diameter increased to ∼80 nm (Fig. 1d) while maintaining a homogeneous appearance. The retention of the negative *ζ*-potential value (−35 mV) indicates that nanoobjects were still covered by SDS with the anionic heads exposed outside. The longest sides for the crystal-shaped objects collected from several TEM images (Fig. S4†) mostly fall in the range of 100–200 nm (Fig. 1h). During the aggregation and shape-shifting processes, the composition and structure of the original cluster were essentially preserved. The absence of chemical reactions was confirmed by the ESI-MS and absorption spectra of the recovered clusters extracted from state III by treatment with dichloromethane (Fig. S5†).

The crystalline nature of the nanocomposites in state III was confirmed by XRD analyses in solution using a synchrotron X-ray source. As shown in Fig. 1i (red line), several moderately sharp peaks were observed, which likely reflect the arrangement of the gold clusters considering the inherent high sensitivity of heavy atoms in X-ray diffraction. The most intense diffraction peak at *q* = 4.29 nm^−1^ corresponds to a lattice-plane distance (*d*) of 14.7 Å, which approximately matches the thickness of 1 (Fig. 1b). Therefore, cluster molecules are likely to form stacked arrays, although it is not easy to propose a 3D arrangement from these data. In contrast, before sonication (state II), the 1–SDS composite showed only a rather broad peak (Fig. 1i, blue line), indicating very low crystallinity. Namely, the cluster aggregates are essentially amorphous in structure at the as-prepared stage (state II) but become crystalline upon sonication, accompanied by distinct sphere-to-rhombus shape shifting (state III). The difference in structure between states II and III was also confirmed by powder X-ray diffraction (PXRD) of the solid samples after evaporation. In addition to the peaks attributed to SDS, only a broad hump was found for the sample derived from the as-synthesized state II, whereas several clear peaks were observed for the state III sample (Fig. S6b *vs.* c†). In this regard, the PXRD profile of the crystalline solid of 1-NO_3_, obtained by simple recrystallization from solution, was different from that of the solid sample from state III (c *vs.* d). Therefore, the packing structure of the nanocrystals in state III is uniquely obtained by nanocrystallization coupled with SDS.

**Fig. 2:**
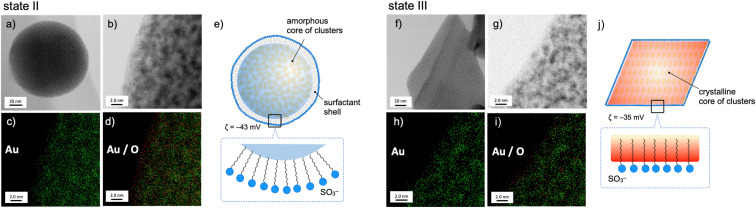
STEM (a, b, f and g) and EDS mapping (c, d, h and i) images, and schematic illustration of the structures (e and j) of 1–SDS composites prepared from 1-NO_3_ (0.03 mM) and SDS (0.20 mM) in MeCN/water (10/90 v/v) before (state II) and after (state III) the 1 h sonication treatment. For the EDS mapping images, (c) and (h) represent the distributions of the Au atoms (green dots) which are overlaid with O atoms (red dots) in (d) and (i). In (d), an O-rich region is seen near the edge of the object, while Au- and O-rich regions are almost overlapped with each other in (i). For (e) and (j), magnified surface structures are schematically shown in the insets.

The nanoscale structures of the above two states of the 1–SDS composites (states II and III) were further studied by scanning transmission electron microscopy (STEM)/energy dispersive X-ray spectroscopy (EDS), and the distributions of the Au and O atoms were mapped. At the rim of the spherical nanoobject of state II (Fig. 2, left), the O atoms resulting from the SO_3_^−^ of SDS are more dominant than the Au atoms (c and d), revealing the presence of a surfactant-rich layer with a width of ∼10 Å, which approximately matches the length of one dodecyl chain. Thus, as schematically illustrated in (e), the SDS monolayer seems to be placed on the surface of the cluster aggregates. On the other hand, for the crystalline objects in state III, no such surfactant-rich zone was observed (Fig. 2h and i); accordingly, the distributions of Au and O atoms mostly overlapped (right). This observation suggests that the dodecyl chains deeply penetrate the cluster-aggregate core (j). On one hand, terminal sulfate anions are considered to still be exposed on the surface of the objects, considering the negative *ζ*-potential (−35 mV, Fig. 1d). Therefore, the shape shifting from state II to state III is considered to involve the migration of the cluster molecules and the packed dodecyl chains (Fig. 2e–j), which may be driven by the electrostatic interactions of the cationic clusters with the distal anionic heads (sulfates) and the hydrophobic effects of the lipophilic segments.

### Critical SDS concentration for nanocrystallization

The amorphous-to-crystalline transition of the 1–SDS composites, thus observed, is considered to be the result of crystallization inside the SDS envelopes. To obtain further insights into the role of SDS, we performed a control experiment in the absence of SDS under otherwise identical conditions (state I′, Fig. 1c). Unlike when coupled with SDS (state III, Fig. 1f), single-crystal-like objects were hardly observed (Fig. 1g). Apparently, sonication caused a certain degree of growth of the spherical aggregates and agglomeration (bridging), but these profiles were not clean. This contrasting result indicates that the crystallization of the cluster molecules is regulated within the SDS envelope. In this regard, the charged amount of SDS was found to be a critical factor dominating the nanocrystallization processes. When the initial SDS concentration ([SDS]_0_) was decreased from 0.20 mM (Fig. 1f and S7c†) to 0.16 mM, the sonicated sample of the composite displayed mostly nanospheres with diameters of ∼50 nm where a few single-crystal objects were observed (Fig. S7b†). At a lower [SDS]_0_ such as 0.14 mM, no crystalline objects were detected (Fig. S7a†) and only agglomerated objects similar to those in state I′ ([SDS]_0_ = 0 mM) (Fig. 1g) were found. On the other hand, when [SDS]_0_ is higher than 0.20 mM (*e.g.*, 0.5, 1.0 and 5.0 mM, Fig. S7d–f†), crystalline materials were predominantly obtained. Therefore, there exists a critical SDS concentration for the nanocrystallization.

It is generally accepted that crystallization starts with the formation of nuclei. The above findings suggest that full coverage of the surface of the original cluster aggregates by SDS, which allows dense packing of the alkyl groups, may facilitate the nucleation of the cluster molecules at the interface. On the other hand, when a limited amount of SDS was applied, namely [SDS]_0_ < ∼0.16 mM in the present case, the packing of the alkyl chains may be loose or allow the formation of defects in the SDS layer, which would deter initial nucleation. In accord with this mechanism, the packing of the linear alkyl chains of the surfactants was found to play a critical role in the nanocrystallization. For example, when the dodecyl (C12) group of SDS was switched to octadecyl (C18) and the mixture was sonicated under standard conditions ([surfactant]_0_ = 0.2 mM, sonication for 1 h), similar nanocrystals were obtained (Fig. S8a†). In contrast, no nanocrystal formation was observed when a decyl analogue (C10) (b). In this relation, it was reported that the critical micelle concentration (CMC) of sodium decyl sulfate is larger by almost one order of magnitude than that of SDS,^32^ indicating that the C10 decyl chain has a much lower packing ability than the C12 dodecyl chain. Therefore, the decyl-chain assembly around the surface of cluster aggregates is considered rather loose. From these observations, it is likely that dense surfactant assemblies are critical for the nanocrystallization, which serve as templates for promoting nucleation to facilitate subsequent growth into nanocrystals.

### Surfactant envelopes as vessels for controlled crystallization

**Fig. 3:**
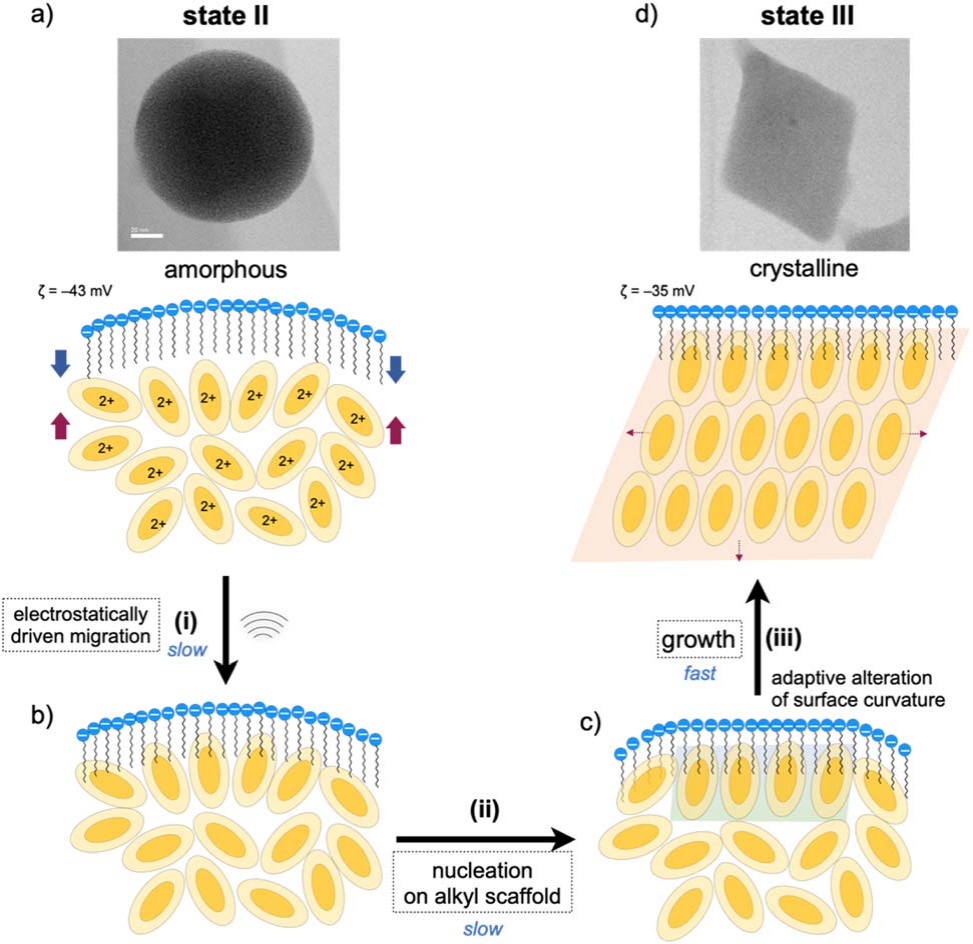
Plausible pathways for the nanocrystallization of the amorphous aggregate of 1 within an SDS envelope (state II (a) → state III (d)). (i) (a) → (b) Sonication-induced penetration of 1 into the dodecyl-chain layer of SDS *via* electrostatic interactions between cationic nanoclusters and anionic sulfate heads and hydrophobic effects. (ii) (b) → (c) Nucleation and self-assembling of clusters at the interface with the SDS layer causing the alteration of the interface curvature. (iii) (c) → (d) Growth from the nucleus within the SDS envelope to afford the nanocrystal. The nitrate ions which accompany the divalent Au_8_ clusters are omitted for clarity.

As mentioned, the amorphous-to-crystalline transition of the 1–SDS composites is induced by the addition of sonication energy. In general, sonocrystallization involves the generation of high-temperature and high-pressure fields (known as ultrasonic cavitation), and the resulting supersaturated situation leads to crystal nucleation.^17,33^ As discussed in the elemental mapping profiles in Fig. 2, crystallization accompanies the penetration of the dodecyl chains of the SDS layer into the interior cluster aggregate. The addition of local thermal energy by sonication may promote the migration of the clusters to the surfactant domains *via* electrostatic interactions and hydrophobic effects (Fig. 3(i)), enhancing the adsorption of the Au_8_ molecules on the alkyl-chain scaffold. In this regard, it has been reported that surfactant assemblies can serve as templates for promoting the nucleation of solute molecules to trigger their growth into crystals,^34,35^ for which a decrease in the surface tension is claimed. Thus, the cluster molecules may prenucleate to self-organize on the SDS scaffold assisted by the inherent polar nature of the Au_8_ framework (Fig. 3(ii)). Subsequently, crystal growth occurs within the SDS envelope from the self-organized clusters at the interface (iii). In the latter two processes (ii and iii), the SDS coating segments may be simultaneously reorganized by adapting to the arrangements of the cluster molecules inside, as schematically shown in Fig. 3c. This process finally leads to spherical-to-angular nanoscale shape shifting as a result of the alteration in the curvature of the nanoobject surface due to the flexible nature of the SDS segment. Thus, the SDS layer is considered to play critical roles not only in nanocrystal formation but also in shape-shifting behavior at the nanoscale. In this connection, when the sonication time was shortened (5 or 30 min) under standard conditions ([SDS]_0_ = 0.2 mM), the TEM images showed only spherical starting objects and nanocrystals, where no transitional species were found (Fig. S9†). From these all-or-nothing profiles, the nucleation and self-organization processes (Fig. 3(i) and (ii)) are considered slow and hence could be the rate-determining steps. Once the crystal seeds are formed by the organization of the cluster molecules at the interface, subsequent crystal growth seems to occur smoothly to give crystalline assemblies (iii). This stepwise mechanism of nanocrystallization agrees well with the above time-course profile.

Finally, we should point out that the DLS and TEM sizes of the spherical and rhombic crystalline nanoobjects, as observed before and after sonication treatment, fall on the same order of magnitude (Fig. 1d–f). This result supports that crystallization essentially occurred in an “*in situ*” manner within the SDS envelope. Thus, the assembled surfactant layer can serve as an efficient and firm barrier to prevent further crystal growth or agglomeration. In fact, the SDS-coated nanocrystals (state III) were very stable in solution, as the features of the TEM image of state III were almost retained even after the solution sample was kept under ambient conditions (r.t., dark) for several months (Fig. S10a†). During crystallization, crystal growth is considered to continue until the free cluster molecules inside are consumed, and further growth may be blocked because the surfactant envelope hinders the release and incorporation of the cluster molecules. In these processes, the flexible nature of the alkyl chains of the surfactant layer also appears to be important and may allow the alteration of the nanoscale sphere-to-rhombus shape shifting in response to the extent of the crystallization of the cluster monomers inside. Thus, the micelle-like architecture composed of SDS serves as a “tough but flexible envelope” to allow specific crystallization within the inner nanospace.

### Crystallization-induced photoluminescence emission enhancement

Aggregation-induced emission (AIE) is a general phenomenon that is frequently found in various luminescent modules.^36–38^ As reported previously, the [core + *exo*]-type Au_8_ clusters used here exhibit unique AIE behaviors; the monomeric form gives a simple excitonic emission (fluorescence) at ∼600 nm, whereas self-assembly under appropriate conditions results in the emergence of a new red-shifted long-lived emission, thus providing an example of a unique type of AIE.^29^ Spectroscopic studies suggested that the AIE originates from minor parts of the clusters that form ordered self-assembled structures. In this respect, it is interesting to see how the nanocrystallization observed in the 1–SDS composites affects the AIE feature. This system provides suitable models for investigating the effects of crystallinity because both nanocrystalline and amorphous forms are available and can be assessed in a single batch.

**Fig. 4:**
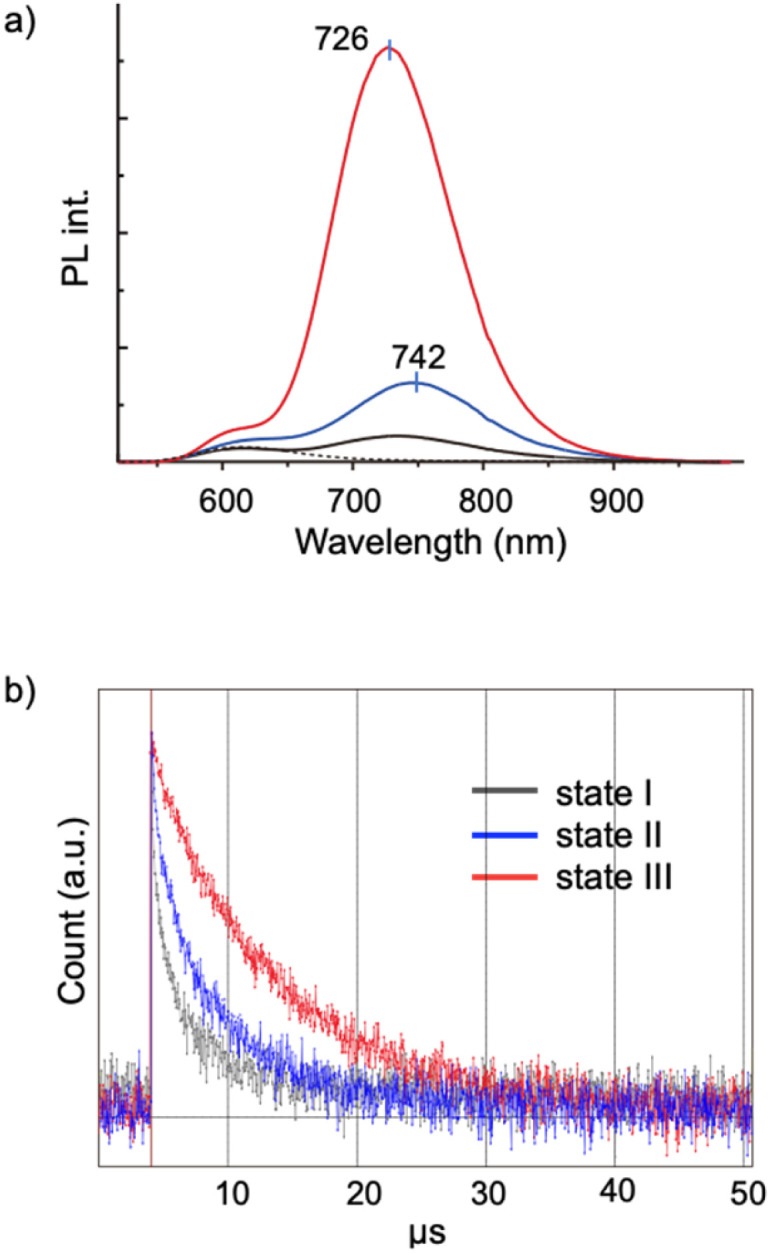
(a) Photoluminescence spectra (*λ*_ex_ = 500 nm, 25 °C) of 1-NO_3_ (0.03 mM) in MeCN (dotted) and in MeCN/water (10/90 v/v) (state I, gray), and of the mixtures of 1-NO_3_ (0.03 mM) and SDS (0.20 mM) in MeCN/water (10/90 v/v) before (state II, blue) and after (state III, red) the 1 h sonication treatment. The shoulders at ∼600 nm found in the spectra of states I, II, and III are due to intrinsic fluorescence of the cluster. (b) Time-resolved decay profiles of states I, II, and III.

Fig. 4a shows the PL spectra of the monomeric and aggregate forms (states I, II and III) of 1. Clearly, the PL emission of the solution of the 1–SDS composite significantly developed upon the transition from the amorphous state (II, blue) to the crystalline state (III, red). The electronic absorption spectra and the absorbance at the excitation wavelength (500 nm) of states II and III were similar to each other, implying that the ground-state electronic structures remained unchanged during nanocrystallization. Although the peak shapes of the PL bands look similar, the peak position underwent a definite blue shift (742–726 nm), indicating an alteration in the energy level of the excited state that is responsible for the emission. The quantum yield (*Φ*) of state III was estimated to be 9.9%, which is nearly four times greater than that of state II (2.5%) and comparable to that of the μm-order microcrystals of 1-NO_3_ obtained by simple recrystallization (10.6%) (Table 1, entry 3 *vs.* 4). Therefore, the emission efficiencies are essentially dependent on the degree of crystallinity, thus providing a clear example of crystallization-induced emission enhancement.^39–44^ It should be noted that the PL emission was very stable, and the intensity was almost retained even after the solution in state III was left under ambient conditions for 3 months (Fig. S10b†), which agrees well with the maintenance of the size and shape of the nanocrystalline state (a). In addition, the quantum efficiency of state II is definitely larger than that of state I, which is formed in the absence of SDS solely by the hydrophobic effects of the phosphine ligands. Thus, the SDS layer surrounding the cluster aggregates in state II may have the intrinsic capability to induce the ordered alignment of the cluster molecules at the interface.

**Table 1:** Absorption and photoluminescence properties (*λ*_ex_ = 500 nm, 25 °C) of 1–SDS composites in MeCN/water (10/90 v/v) and single crystals of 1-NO_3_ in the solid state

Entry	State	*λ*_abs_	*λ*_PL_	*Φ* (%)	*τ* (μs)	*k*_r_ × 10^3^ (μs^−1^)	*k*_nr_ (μs^−1^)
1	I	535	740	0.57	1.9	2.9	0.51
2	II	529	742	2.5	3.9	6.3	0.25
3	III	529	726	9.9	6.8	14.6	0.13
4	μm-crystals	513	727	10.6	14.7	7.2	0.061

The PL properties of the aggregates were further investigated in terms of the PL lifetime. As seen from the decay profiles shown in Fig. 4b, the emissive excited states of nanocrystalline state III decay slower than those of amorphous state II, with lifetimes (*τ*) of 6.8 and 3.9 μs, respectively (Table 1, entries 2 and 3). Taken together with the *Φ* values, the rate constants for the radiative (*k*_r_) and nonradiative (*k*_nr_) paths were calculated, which reveal that the enhancement of crystallinity results in an increase in the probability of the radiative path. Thus, when compared in the amorphous assembly (state II), the cluster molecules in crystalline state III should be packed rather tightly as a result of the restriction within the crystalline lattices. This may cause the effective suppression of the non-radiative path, leading to the enhancement of the quantum efficiency (*Φ*). It should be also noted that there are definite differences in the decay kinetics between the nanocrystals (state III, in solution) (entry 3) and the μm-crystals (in the solid state) (entry 4), albeit they gave comparable quantum efficiencies (*Φ* = ∼10%); the emission of the microcrystals of 1-NO_3_ had a longer lifetime and lower *k*_r_ and *k*_nr_ values. These differences may originate from the nature of the excited state responsible for the radiative path. Therefore, the ordering of the cluster molecules may induce the alteration of the energy level and dynamic properties of excited electrons. In addition, we must consider other factors such as crystal size and morphology. In this respect, it would be interesting to establish methods to control the size and morphology at the nanoscale by careful tuning of the nanocrystallization conditions. Comprehensive investigations of the nanostructure and the photodynamic properties could further elucidate of the AIE behaviors of the present Au_8_ nanocluster as a unique type of luminogen.

## Conclusions

In this paper, we have provided a successful example of the solution synthesis of single nanocrystals of gold nanoclusters by using surfactant-based nano-vessels. Giant spherical micelles encapsulating an amorphous cluster aggregate inside were exclusively converted into rhombic nanocrystals upon simple sonication treatment. The observed sphere-to-rhombus shape shift is likely a result of the “*in situ*” crystallization event within the surfactant nano-envelopes since no single nanocrystals were obtained in the absence of the surfactant additives. The robust but flexible nature of the surfactant-based nano-envelope may contribute to controlled nanocrystallization: robustness prevents unfavourable uncontrolled growth, agglomeration, and precipitation, while flexibility allows sphere-to-rhombus shape-shifting at the nanoscale. The robustness of the surfactant wall also results in the excellent stability of the nanocrystals in solution. These findings imply the utility of surfactant-based inner nanospaces for the facile synthesis of molecular nanocrystals. The present method may be applicable to various molecules, expanding the horizons of nanosized molecular crystals. We have also taken advantage of the amorphous-to-crystalline transition in homogenously dispersed solutions and provided solid proof that the increase in crystallinity is closely associated with the AIE intensity. This is a definite example of ‘crystallization-induced emission enhancement’. This work is the first example showing a facile synthetic method and unique properties of nanocrystals of metal nanoclusters, which could lead to new perspectives in the field of nanocluster science. The remaining issue is the atomic structure-based elucidation of the crystallization processes as well as the photophysical properties. The recently developed microED (microcrystal electron diffraction) method,^45^ which involves the collection of electron diffraction data during TEM measurements and has recently emerged as a state-of-the-art analytical technique suitable for the structural determination of ultrasmall crystals, is promising and worthy of further investigation.

## Data availability

The data supporting this article have been included as part of the ESI.†

## Author contributions

YSA conducted experimental investigations and wrote the original draft of the paper. AY, MS, SN, HN conducted experimental investigations. YSH checked the data and manuscript. YI conducted synchrotron XRD experiments and checked the data and manuscript. KK supervised and conceptualized the research, acquired financial support, and wrote the paper.

## Conflicts of interest

There are no conflicts to declare.

## Supplementary Material

SC-015-D4SC02834A-s001
