# Supplementary material for: Controlled nanocrystallization of gold nanoclusters within surfactant envelopes: enhancing aggregation-induced emission in solution
Source: Chem Sci. 2024 Jun 24;15(30):11775–82. doi: 10.1039/d4sc02834a (PMC11290417; doi:10.1039/d4sc02834a)
Supplement: SC-015-D4SC02834A-s001 [file SC-015-D4SC02834A-s001.pdf]

Electronic Supplementary Material (ESI) for Chemical Science.
This journal is © The Royal Society of Chemistry 2024

Electronic supplementary information

Controlled nanocrystallization of gold nanocluster within surfactant envelopes enhancing aggregation-induced emission in solution

Yuki Saito, Ayano Suda, Maki Sakai, Shogo Nakajima, Yukatsu Shichibu, Hayato Kanai, Yasuhiro Ishida,* Katsuaki Konishi *

Graduate School of Environmental Science, Hokkaido University, North 10 West 5, Sapporo 060-0810 Japan. Faculty of Environmental Earth Science, Hokkaido University, North 10 West 5, Sapporo 060-0810 Japan. RIKEN Center for Emergent Matter Science, 2-1 Hirosawa, Wako, Saitama 351-0198, Japan

METHODS

General

Tetrachloroauric(III) acid tetrahydrate (>47.5% for gold) was obtained from Tanaka Kikinzoku Kogyo. 1,3-Bis(diphenylphosphino)propane (dppp) (Tokyo chemical industry), sodium dodecyl sulfate (SDS) (FUJIFILM Wako) and potassium cyanide (FUJIFILM Wako) were used as received. Other standard chemicals and solvents were purchased from Kanto chemical and used as received. Dynamic light scattering (DLS) and ζ-potential data were collected on a Malvern Zetasizer Advance-Pro (Red) light scattering system with a 633 nm He/Ne laser at 20 °C using a glass cuvette for DLS and a plastic cell for the ζ-potential measurement, respectively. Transmission electron microscopy (TEM) images were obtained by JEM-2000FX at Faculty of Engineering, Hokkaido University. Scanning transmission electron microscopy (STEM) images and energy dispersive X-ray spectroscopy images were obtained on a JEOL JEM-ARM200F at RIES, Hokkaido University. X-ray diffraction data in solution were obtained at BL05XU in SPring-8 (Hyogo, Japan). Diffraction data were collected using Dectris model Pilatus 3S 1M detectors. The incident X-ray beam (1.00 Å wavelength) was monochromated by a Si (111) double-crystal monochromator. The sample-to-detector distance was 249.8343 mm. The scattering vector *q* and the position of an incident X-ray beam on the detector were calibrated using several orders of layer reflections from silver behenate. A solution sample was held in a 1.5 mm-diameter glass capillary exposed to an X-ray beam for 1.0 s at 25 °C. Powder X-ray diffraction (PXRD) profiles were obtained at room temperature on a Bruker D2 PHASER 2nd Generation with CuKα radiation. The powder samples of **1**-SDS composites (state II and III) were prepared by solvent evaporation using a freeze-drying machine, and were directly subjected to the measurement. Visible absorption and photoluminescence spectra were recorded at 25°C on a JASCO V-670 and FP-8600 spectrometers, respectively, using a quartz cell with a 1-cm path length. Photoluminescence spectra were corrected with rhodamine B and a JASCO ESC-333 standard light source unit, and the quantum yields were determined relative to rhodamine 6G in ethanol. Lifetime data were collected on a Hamamatsu photonics Quantaurus-Tau with the excitation wavelength at 280 nm. Electrospray ionization mass (ESI-MS) spectra were recorded on a Bruker microTOF-HS. Sonication treatments were performed on an SND US-102 ultrasound water bath.

Synthesis of $[Au_8(dppp)_4(CN)_2](NO_3)_2$ (1-NO$_3$)

The precursor cluster $[Au_8(dppp)_4](NO_3)_2$ was synthesized by previously reported procedure.[26] To a 2-necked round-bottomed flask, $[Au_8(dppp)_4](NO_3)_2$ (71 mg, 22.0 μmol) and methanol (30 mL) were added, and then 2.8 mL of methanol solution of potassium cyanide (1 mg/mL, 44.0 μmol) was added to the flask. During the 12-h stirring under room light at

room temperature, the color of the reaction solution changed from violate to pink. Then, 180 mL of diethyl ether was added to the flask and the resulting precipitates were filtered through a membrane filter and washed with diethyl ether (10 mL × 3), and then dried in vacuo. The crude cluster was further purified by recrystallization by the vapor diffusion method using methanol/dichloromethane = 50/50 as the good solvent and diethyl ether as the poor solvent to give $[Au_8(dppp)_4(CN)_2](NO_3)_2$ (38 mg, 27% yield) as pink crystals. Vis (MeOH) : λ_{max} 524.0 nm. ESI-MS (MeOH): calcd; 1638.2; obsd; 1638.1. The similarity of the visible absorption spectral pattern to those of the fully characterized [core+*exo*]-type Au_8 clusters[26,27,29,31] coupled with the above ESI-MS result (**Fig. S1**) supports the structure of the cluster moieties shown in **Fig. 1b**. This molecular structure of the cluster was confirmed by preliminary SCXRD studies.

Nanocrystallization of Au_8 cluster in solution

An aqueous solution of SDS (5 mM, 120 µL) and $[Au_8(dppp)_4(CN)_2](NO_3)_2$ (**1**-NO_3) in acetonitrile (0.3 mM, 300 µL) were mixed in an 8 mL vial and then diluted with Milli-Q water (2580 µL). The final concentration of the Au_8 cluster and SDS were designated to be 30 µM and 200 µM, respectively. The solution was then sonicated for 60 min using a 38 kHz ultrasound bath. During sonication, the water temperature in the bath was maintained between 25-35 °C.

Preparation of TEM and STEM samples

The sample solutions were diluted two-fold with the mixed solvent (MeCN/water = 10/90), and then the diluted solution was deposited on a TEM grid (Nisshin EM, Fine grid, Cat. 653, 200 mesh, Cu). All grids were dried in vacuo for 24 hours and then subjected to the TEM and STEM measurements.

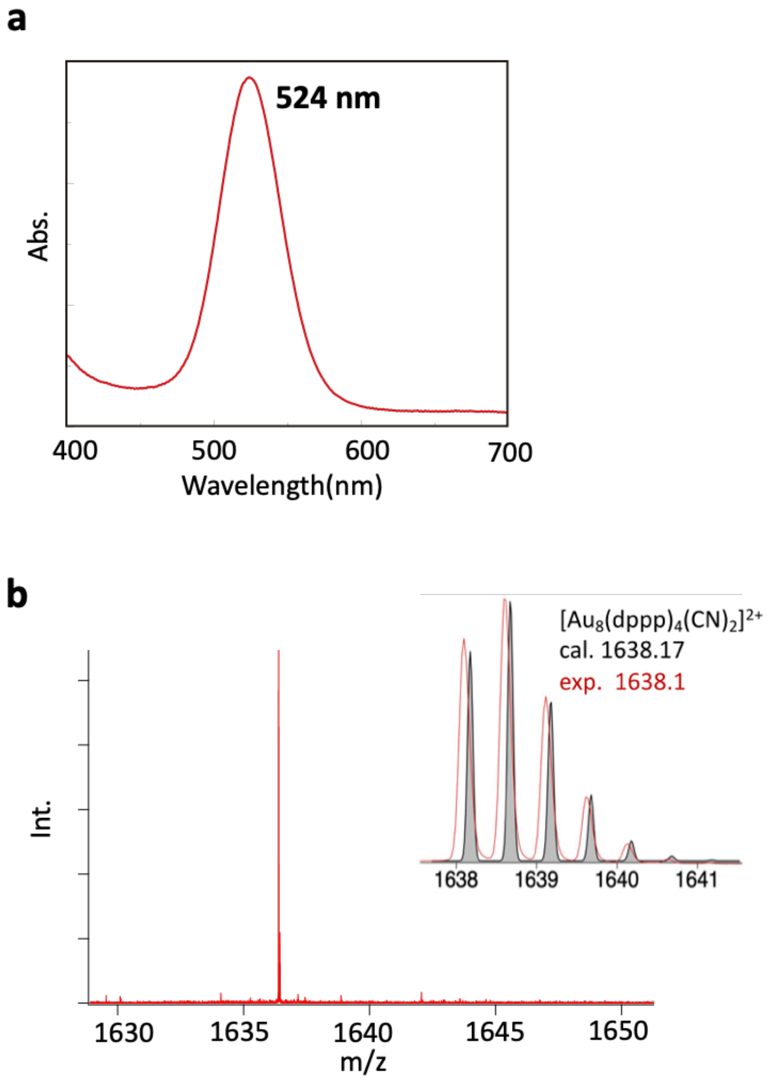

Fig. S1. (a) Absorption and (b) ESI-MS spectrum of the nitrate salt of $[Au_8(dppp)_4(CN)_2]^{2+}$ (**1**).

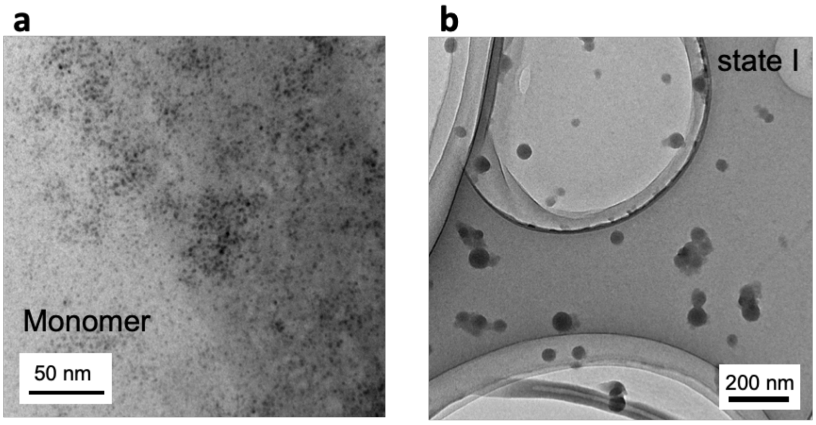

Fig. S2. TEM images of the samples prepared from the solutions of the nitrate salt of **1** in (a) MeCN alone (monomer) and (b) MeCN/water (10/90 v/v) (**state I, Fig. 1c**).

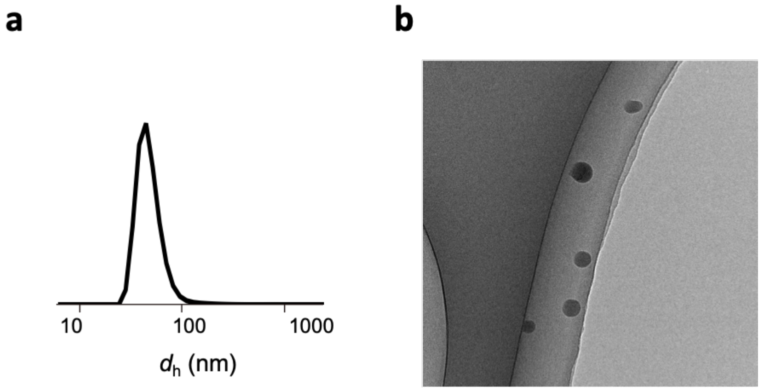

Fig. S3. TEM image and DLS profile of the solution sample of **state II** after storage under ambient conditions for 5 days.

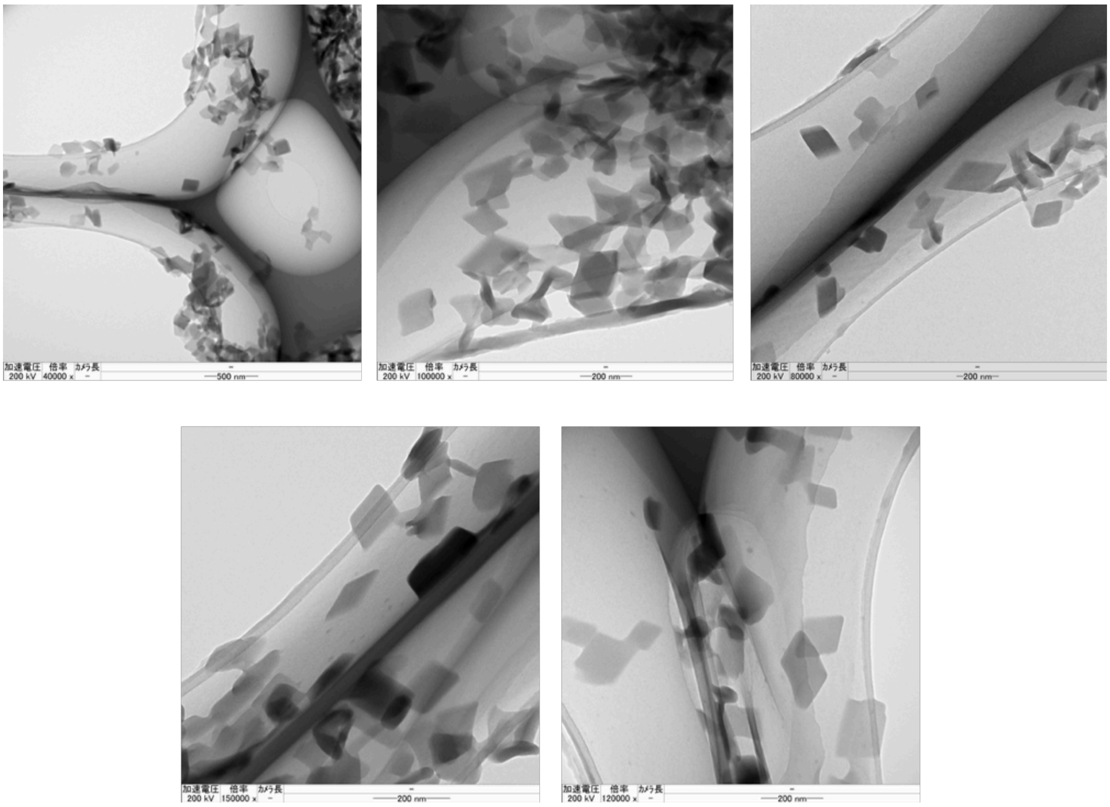

Fig. S4. TEM images of **state III**.

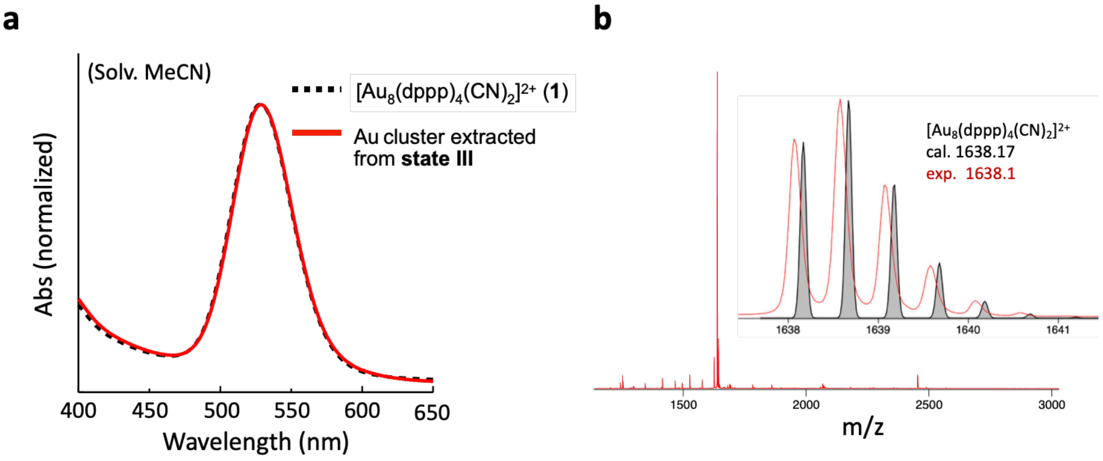

Fig. S5. (a) Absorption (b) ESI-MS spectra of the Au cluster extracted from the solution sample of **state III** in MeCN/water with dichloromethane. For (a), the spectrum of the original cluster (**1**) is overlaid for reference.

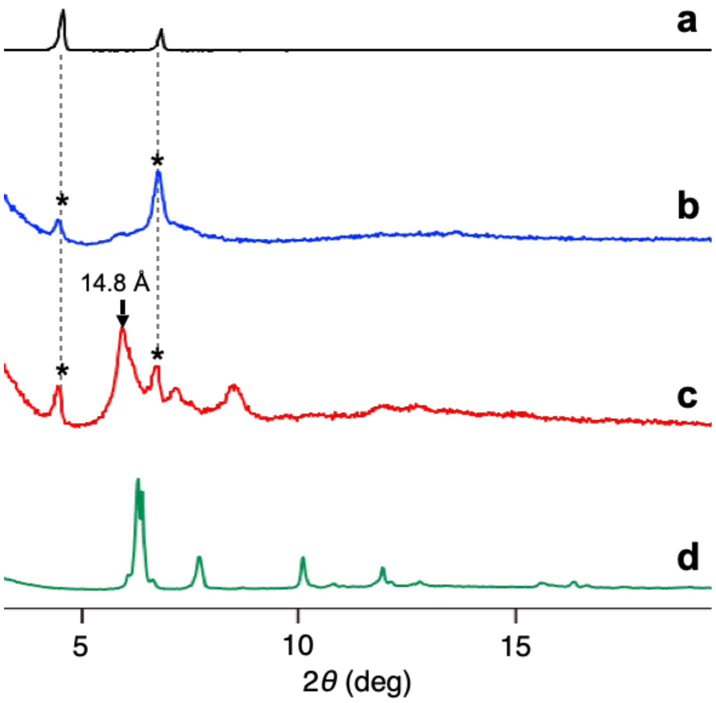

Fig. S6. PXRD profiles of solid samples of (a) SDS alone, **1**-SDS composites (**b state II**; **c state III**, see Fig. 1c) prepared from the solutions of MeCN/water (10/90 v/v) by solvent evaporation, and (d) single crystals of the nitrate salt of **1** obtained by recrystallization from CH_2Cl_2/CH_3OH/ether. Asterisks in (b) and (c) indicate the peaks due to the lamellar structure of SDS.

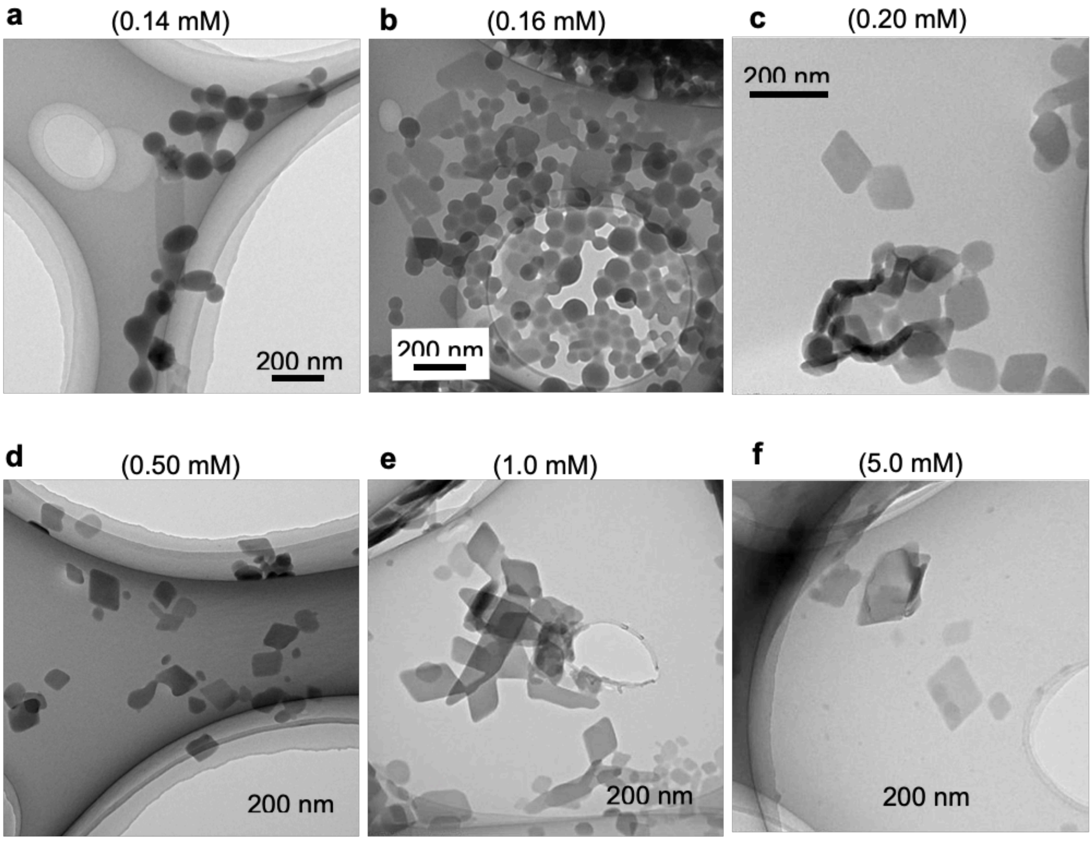

Fig. S7. TEM images of the samples of **1** (0.03 mM) upon sonication for 1 h in MeCN/water (10/90 v/v) in the presence of SDS of varying initial concentrations (a-f). The initial concentration of SDS is given above each image in parenthesis.

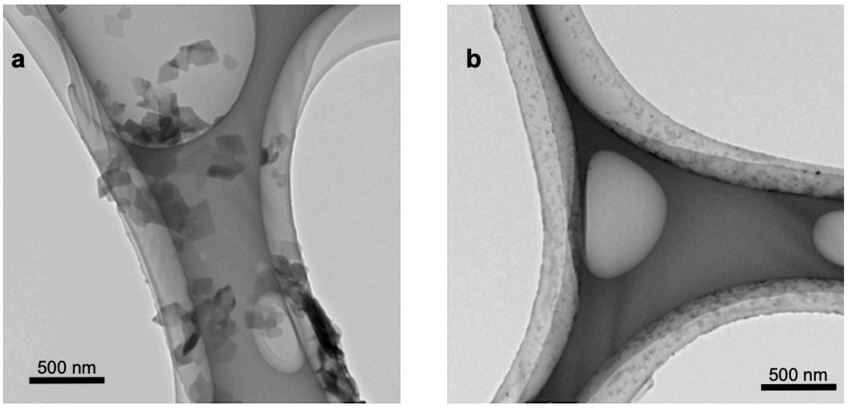

Fig. S8. TEM images of the composites prepared from the solution of **1**-NO_3 in MeCN/water (10/90 v/v) containing (a) sodium octadecyl sulfate and (b) sodium decyl sulfate ([surfactant]$_0$ = 0.20 mM) upon sonication for 1 h.

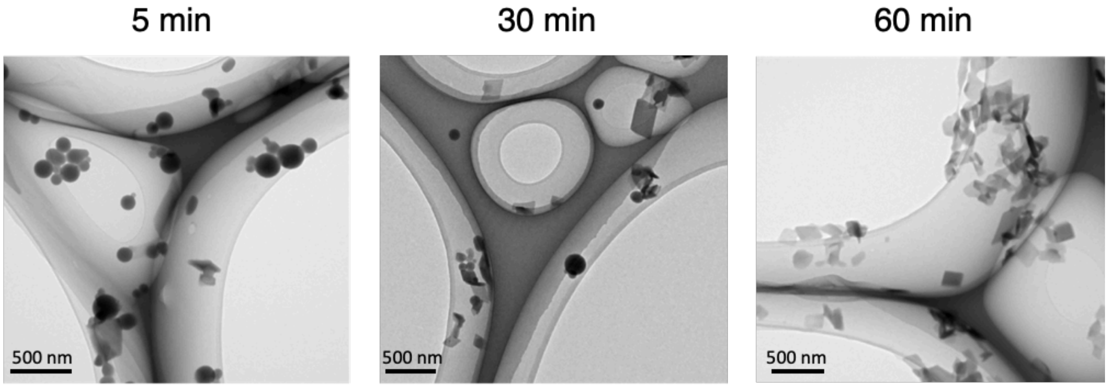

Fig. S9. TEM images of sonication-induced nanocrystallization of **1**-SDS composite with varying sonication times ([**1**]$_0$ = 0.03 mM; [SDS]$_0$ = 0.20 mM) in MeCN/water (10/90 v/v).

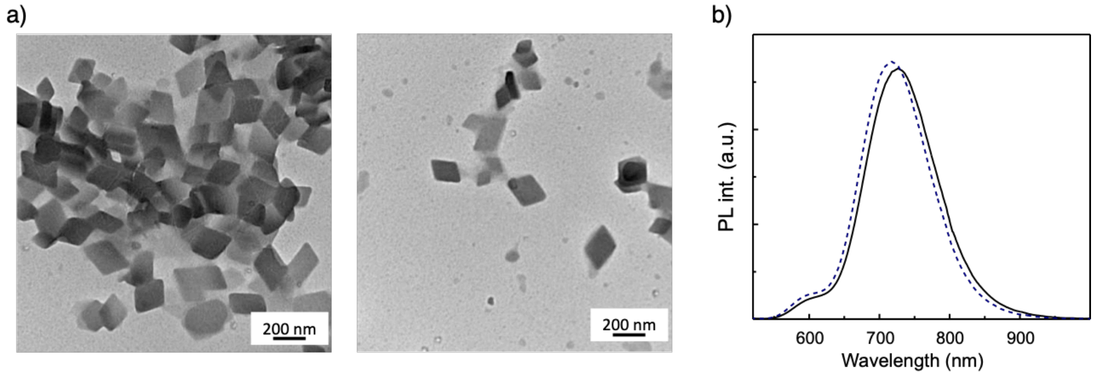

Fig. S10. a) TEM images of the sonicated **1**-SDS composite ([**1**]$_0$ = 0.03 mM; [SDS]$_0$ = 0.20 mM; sonication time = 1 h) after being stored under ambient dark conditions for 3 months. b) Photoluminescence spectra of **1**-SDS composite just after the sonication treatment (solid line) and the same sample after being stored under ambient dark conditions for 3 months (dashed line).
